# Hyperactivity precedes conduct problems in preschool children: a longitudinal study

**DOI:** 10.1192/bjo.2018.20

**Published:** 2018-06-22

**Authors:** Berit M. Gustafsson, Henrik Danielsson, Mats Granlund, Per A. Gustafsson, Marie Proczkowska

**Affiliations:** Center for Social and Affective Neuroscience, Department of Clinical and Experimental Medicine, Linköping University, Sweden, Psychiatric Clinic, Högland Hospital, Division of Psychiatrics and Rehabilitation/Region Jönköping, Sweden, and CHILD research environment, SIDR, Jönköping University, Sweden; Swedish Institute for Disability Research and Department of Behavioural Sciences and Learning, Linköping University, Sweden; CHILD research environment, SIDR, Jönköping University, Sweden and Department of Special Education, Oslo University, Norway; Center for Social and Affective Neuroscience, Department of Clinical and Experimental Medicine, Linköping University, Sweden; and Department of Child and Adolescent Psychiatry, Linköping University, Sweden; Center for Social and Affective Neuroscience, Department of Clinical and Experimental Medicine, Linköping University, Sweden, and Psychiatric Clinic, Hospital of Jönköping, Division of Psychiatrics and Rehabilitation/Jönköping County, Sweden.

## Abstract

**Background:**

Externalising problems are among the most common symptoms of mental health problems in preschool children.

**Aims:**

To investigate the development of externalising problems in preschool children over time, and the way in which conduct problems are linked to hyperactivity problems.

**Method:**

In this longitudinal study, 195 preschool children were included. Latent growth modelling of conduct problems was carried out, with gender and hyperactivity at year 1 as time-invariant predictors.

**Results:**

Hyperactivity was a significant predictor for the intercept and slope of conduct problems. Children with more hyperactivity at year 1 had more conduct problems and a slower reduction in conduct problems. Gender was a significant predictor for the slope of conduct problems.

**Conclusions:**

Children with more initial hyperactivity have less of a reduction in conduct problems over time. It is important to consider the role of hyperactivity in studies of the development of conduct problems.

**Declaration of interest:**

None.

It is important to pay attention to early signs of mental health problems in order to prevent secondary problems later in life.^1^ Externalising problems are among the most common symptoms of mental health problems in young children,^2^ and in preschool children they are frequently operationalised to include both hyperactivity and conduct problems.^3^ In more difficult cases, these behaviours can be predictors for symptoms in the same or overlapping domains,^4^ or for psychiatric symptoms years later.^5^ Previous research has shown that some children who have a high activity level, impulsivity and inattentive behaviour at preschool ages not only tend to show persistent problems in the same area,^6^ but also have problems in other mental health domains, such as conduct problems, poor social functioning and dysfunctional emotional regulation.^7^

## Externalising problems

Hyperactivity is relatively stable over time for preschool children. Previous studies also show that boys show a higher degree of hyperactivity than girls.^8^^,^^9^ Attention-deficit hyperactivity disorder (ADHD) symptoms, such as hyperactivity, tend to decline with age, but up to 65% of children aged 4–12 years with ADHD will still experience impairing symptoms in adulthood;^10^ in other words, ADHD can be a chronic and often lifelong disorder. There is a high rate of comorbidity for oppositional defiant disorder (ODD) and ADHD.^11^

Earlier studies have investigated the relationship between hyperactivity and conduct problems regarding symptoms in childhood and adolescence.^12^ Taylor *et al*.^13^ have shown that hyperactivity in childhood could eventually lead to conduct problems in adolescence. Burns and Walsh found that ADHD symptoms influenced the development of ODD behaviours among school children.^14^ The findings of Harvey *et al*^15^ for preschool children support the notion that ADHD may be causally related to ODD, but not *vice versa*.

The preschool years are characterised by the transition from infancy to elementary school age, in which children should increasingly modulate their behaviour appropriately, both in and outside the home.^16^ Improvement of self-regulation in children 4–6 years old has had positive results on behavioural problems later in childhood.^17^ Wahlbeck^18^ has claimed that there is good evidence that prevention achieves the best results if it starts in early childhood, when there may be fewer complications and comorbidities to be treated than is the case with older children. One reason for this is brain plasticity in early childhood.^19^ This approach requires behaviour problems to be identified among young children who are still not diagnosed but show externalising problems. Intervention and prevention should focus on children that show severe externalising behaviours, because they are the most likely to suffer from adult psychopathology.^20^

## Screeening

Different screening instruments can be used for a more structured identification of children with externalising behaviour problems. In activities in preschool with a specific goal or rules, such behaviours are probably more visible. Swedish preschool children have more free play and fewer lesson-like activities in preschool than many other OECD (Organisation for Economic Co-operation and Development) countries,^21^ and thus externalising behaviours may be less frequently observed than in environments characterised by structured, teacher-led activities.^22^ Externalising problems can therefore more easily be observed and identified, and also often handled, in structured activities by preschool teachers in Swedish preschools. The strongest predictor for obtaining special measures is if a child disrupts the preschool group and the preschool teacher's educational work. The Strengths and Difficulties Questionnaire (SDQ) – preschool version – has been validated for identifying behaviour problems in preschools in Sweden.^23^ The SDQ has specific subscales to screen for hyperactivity and conduct problems, respectively. In this study, we describe the early symptoms of hyperactivity and conduct problems according to the SDQ, not diagnoses such as ODD or ADHD.

The present study is part of a longitudinal project, with three yearly waves, studying preschool children's mental health and functioning in the preschool setting.^9^ In this study, we examine how conduct problems develop over time and whether hyperactivity is associated with that development. We made the assumption that hyperactivity would be relatively stable over time^8^ and therefore used hyperactivity as a time-invariant predictor. A clear understanding of how these groups of externalising behaviours relate to each other may be of great help in the preschool setting when special measures are planned for the individual child with problems.

## Aims

The aims of this study were to investigate the development of externalising problems in preschool children over time, and the ways in which conduct problems are linked with hyperactivity problems.

## Method

### Procedure

Preschools from a stratified sample of various-sized Swedish municipalities were invited to participate. The preschool managements in the various municipalities were contacted and consent was requested for participation of their preschool units. The preschool teachers then asked all parents for their written informed consent. Answers were obtained from all the preschool teachers with professional knowledge of the child. Teachers were required to have known the child for at least 6 months, and were asked to base their ratings on at least the 2 preceding weeks.^9^

### Participants

The number of children that participated in all 3 years (2012–2014) was 195; 56% (110) boys and 44% (85) girls. The mean age during the first year of participation was 32 months (s.d. = 9, range 15–57), that during the second year was 44 months (s.d. = 9, range 24–69) and that during the third year of participation was 55 months (s.d. = 9, range 36–71). The participation rate and gender distribution were similar for children of different ages; 23.3% of the children had a mother tongue other than Swedish and 3.8% were officially judged to be in need of special support.

In the first year, 1615 children were invited to participate; of these, the parents of 663 (41.6%) gave their consent. Preschool teachers completed the SDQ for 651 (40%) children, 195 (12%) of whom were included in the study at all three data collection points with complete SDQ forms. Not enough children were included longitudinally to make it possible to include other factors that might moderate and mediate outcome. Of the 456 children who did not participate in all 3 years, 281 (17%) children were in the older age group (48–72 months) that finished preschool and continued to preschool class within another unit, while 175 (11%) children were in the age group 15–48 months and could have participated all 3 years, but could not be followed up because they had changed preschool or because the preschool teachers could not answer the questionnaires owing to their work situation in year 2 and/or year 3. In this group of younger children (11%), significantly more had a mother tongue other than Swedish (34%) compared with the group that participated.

### Instruments

#### Strengths and Difficulties Questionnaire

The SDQ is a 25-item questionnaire measuring child behaviours. It can be used by parents or teachers, or as a self-report by older children.^24^ The SDQ has been translated into Swedish and validated for parental use for children aged 6–10 years, and it has demonstrated good psychometric properties.^25^ The SDQ has been confirmed as having satisfactory psychometric properties to identify 3–4-year-old children with emotional and behavioural difficulties.^26^

In this study, the SDQ teacher version for children aged 2–4 years was used, including the impairment supplement.^27^ The SDQ teacher version has been shown to have satisfactory psychometric properties to identify children with emotional and behavioural difficulties.^23^

The items are divided into five subscales with five items in each subscale, generating scores for emotional symptoms, conduct problems, hyperactivity, peer relationship problems and pro-social behaviours. In an earlier report using data from the first wave of this longitudinal study, we found that the SDQ was a valid instrument for use by teachers in a preschool setting to identify early signs of distress or behaviour problems in young children.^23^ In children of less than 4 years of age, the hyperactivity and conduct problems subscales worked well. In the age group 4–5 years, all four original SDQ problem subscales were tested and produced good results.^23^

In this study, the hyperactivity and conduct subscales of the SDQ teacher version for children aged 2–4 year was used. The five hyperactivity variables were: ‘Restless, overactive, cannot stay still for long’, ‘Constantly fidgeting or squirming’, ‘Easily distracted, concentration wanders’, ‘Can stop and think things out before acting’ and ‘Sees tasks through to the end, good attention span’. The five conduct problems variables in SDQ were: ‘Often has temper tantrums or hot tempers’, ‘Generally obedient, usually does what adults request’, ‘Often fights with other children or bullies them’, ‘Often argumentative with adults’ and ‘Can be spiteful to others’. Each item was scored on a three-point scale: not true; somewhat true; and certainly true. An expert panel consisting of five experienced preschool teachers evaluated each item for its relevance through a consensus discussion. Since the sample included different developmental ages, a question about relevance (yes/no) for the specific child was included after each SDQ item. Overall, most of the items were considered relevant and possible to rate for both younger and older preschool children.^23^

### Statistical analysis

In order to examine how children's conduct problems changed over time, with and without hyperactivity problems, we used latent growth modelling (LGM) within a structural equation modelling framework with IBM SPSS AMOS 23. LGM is mainly applicable because it can model changes in the growth of variables over time, using longitudinal data. LGM models are developed in two steps. The first step (unconditional model) estimates the intercept and slope of the growth curve of the measured variable, repeated at several time points. The second step (conditional model) allows time-invariant predictors to influence the intercept and slope obtained in the first step.^28^^,^^29^

In LGM, the intercept is a constant for each individual over time; hence, the paths from the repeated measures to the intercept have fixed values of 1. The slope represents the linear growth, and therefore the paths to the slope were fixed at 0, 0.5, and 1, respectively, for each repeated measure. Additional model constraints were equal variance for conduct at all time points and allowing covariance between slope and intercept.

For the missing variables in the SDQ subscales for conduct problems (five items) and hyperactivity (five items), at three time points each, the AMOS module for data imputation was used with the regression imputation method. The imputation was constrained so that if any of the five items constituting the conduct problems or the hyperactivity scale was missing, the imputation was only based on variables from the same subscale at the same time point. There is no agreed set of fit indices for reporting LGM results, but recommendations suggest using a variety of indices.^30^ In the present article, the following indices were used as criteria: *P* > 0.05 for the χ^2^ test, meaning that (the covariance matrix of) the model is not significantly different from (the covariance matrix of) the data; comparative fit index and Tuker–Lewis index values > 0.95,^31^ and root mean squared error approximation index < 0.07.^32^ Besides meeting these criteria, all coefficients in the model had to be significant (*P* < 0.05) for the model to be accepted. If several models were acceptable, the model including the most predictors was selected as the best.

### Ethical considerations

This study was approved by the Regional Ethical Review Board in Linköping (Dnr 2012/199–31). Preschool management, preschool teachers and both parents of each child provided written informed consent. All questionnaires were coded and the coding key was kept separate from the questionnaires after the data was collected. If preschool teachers identified children with previously unknown mental health problems in the course of the study, they were instructed to refer them to child healthcare for support.

## Results

### The hyperactivity and conduct problem variables

The present study investigated the trajectory of conduct problems in preschool children over time and how their presentation is linked to hyperactivity problems. Means, s.d. values and correlations for hyperactivity at time point 1 (T1) and conduct problems at time points 1, 2 and 3 (T1, T2 and T3, respectively) can be found in Table 1. A visual inspection showed that conduct problems decreased approximately linearly over time: the mean (s.d.) values were 0.39 (0.43) at T1, 0.33 (0.45) at T2 and 0.24 (0.38) at T3. Conduct problems at these different time points correlated with each other, indicating that an LGM approach is appropriate.

**Table 1:** Mean and s.d. by each variable for conduct problems and hyperactivity and correlations (Pearson) between the variables used in the modelling for the sample

			Correlation [95% CI]		
Variable	Mean	s.d.	1	2	3
T1 conduct problems	0.39	0.43			
T2 conduct problems	0.33	0.45	0.39**		
			[0.26, 0.50]		
T3 conduct problems	0.24	0.38	0.27**	0.58**	
			[0.14, 0.40]	[0.47, 0.67]	
Hyperactivity T1	0.59	0.50	0.59**	0.42**	0.30**
			[0.49, 0.67]	[0.29, 0.53]	[0.16, 0.42]

**P < 0.01.

Previous reports suggest that hyperactivity problems are relatively stable over time.^8^ The mean (s.d.) values for hyperactivity in this study were 0.59 (0.50) at T1, 0.56 (0.57) at T2 and 0.46 (0.51) at T3, showing that hyperactivity was relatively stable between T1 and T2 and that there was not a linear decrease over time. To further investigate whether hyperactivity was stable over time, an LGM model for hyperactivity problems was estimated. The suitability of the model was not satisfactory on all the fit criteria (Table 2) and, more importantly, the slope was not significant. This confirmed that hyperactivity problems were relatively stable over time in this sample; therefore, hyperactivity at T1 was used as a time-invariant predictor in the modelling.

**Table 2:** Fit indices for the latent growth models (LGMs); longitudinal data over 3 years

Model	d.f.	χ^2^(2)	*P*	TLI	CFI	RMSEA
LGM conduct problems	2	0.485	0.785	1.046	1.000	0.000
LGM hyperactivity	4	10.331	**0.035**	**0.931**	0.954	**0.090**
LGM conduct problems hyperactivity predictor	4	1.486	0.829	1.033	1.000	0.000
LGM conduct problems gender predictor	3	0.505	0.918	1.080	1.000	0.000
LGM conduct problems age predictor	3	9.054	**0.029**	**0.807**	**0.942**	**0.102**
LGM conduct problems hyperactivity and gender predictor	7	2.707	0.911	1.032	1.000	0.000

TLI, Tuker–Lewis index; CFI, comparative fit index; RMSEA, root mean squared error approximation index. Bold text indicates values that were not acceptable.

### Latent growth modelling

The unconditional model with conduct problems was evaluated at three time points (T1, T2 and T3), and the suitability of the model was satisfactory on all the fit criteria (Table 2). This meant that the unconditional model could be used as a base for the conditional model in the next step. The model had a significant slope showing that conduct problems decrease over time. A negative covariance between intercept and slope was observed; this was interpreted to mean that children with more conduct behaviour at T1 also had less of a decrease in conduct problems.

The next step in the modelling was to test whether time-invariant predictors could improve the model. Three time-invariant predictors were, in separate models, added to the unconditional model. Hyperactivity at T1 had significant paths to both intercept and slope for conduct. Gender had a significant path to the slope of conduct, but not the intercept. Age at T1 had no significant path to either intercept or slope and was therefore not included in further modelling.

**Fig. 1:**
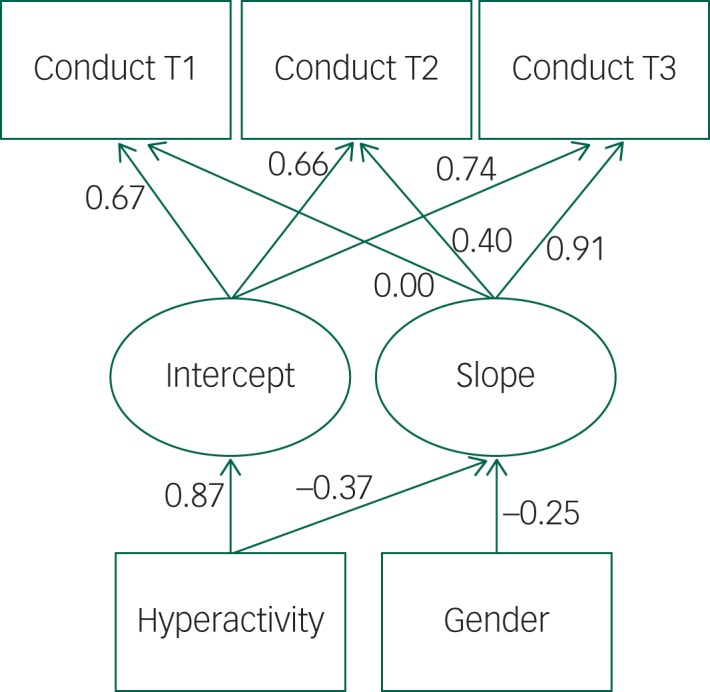
Conditional model for conduct problems over three years with hyperactivity and gender at T1 as time-invariant predictors showing that hyperactivity affects both intercept and slope, and gender affects the slope.

The final model was fitted by adding both hyperactivity and gender as time-invariant predictors in the same model. This final model had the same paths from the time-invariant predictors as the previous models (hyperactivity influenced both the intercept and slope, whereas gender only was associated with the slope). The suitability of the conditional model was satisfactory on all fit criteria (Table 2). An overview of the results can be seen in Figure 1, and all the relevant details (unstandardised and standardised coefficients for paths, covariances and variances) for the final model are presented in Table 3.

**Table 3:** Unstandardised and standardised coefficients (estimates) for paths, means, covariances, correlations and variances for the conduct problems and hyperactivity model with gender

	Part of model			LGM conduct problems, hyperactivity and gender predictor	
				Unstandardised	Standardised
Paths^a^					
	Intercept	←	T1 hyperactivity	0.50	0.87
	Slope	←	T1 hyperactivity	−0.26	−0.37
	Slope	←	Gender	−0.18	−0.25
	T1 conduct problems	←	Intercept	1.00	0.67
	T1 conduct problems	←	Slope	0.00	0.00
	T2 conduct problems	←	Intercept	1.00	0.66
	T2 conduct problems	←	Slope	0.50	0.40
	T3 conduct problems	←	Intercept	1.00	0.74
	T3 conduct problems	←	Slope	1.00	0.91
Means				
	Intercept		0.10	
	Slope		0.27	
	T1 hyperactivity		0.59	
	Gender		1.46	
Variance				
	T1 hyperactivity		0.25	
	Gender		0.25	
	Error T1 conduct problems		0.10	
	Error T2 conduct problems		0.11	
	Error T3 conduct problems		0.01	
	Intercept		0.02	
	Slope		0.10	

a ← denotes a path in the model.

In summary, there were four models with acceptable fit on all indices: the unconditional LGM and three LGMs with different predictors (hyperactivity, gender, and both hyperactivity and gender). As outlined in the Method section, the model with most predictors was selected as the best model. It is described further below.

In the final model, there was no covariance between slope and intercept, and hyperactivity in T1 was positively associated with the intercept, meaning that more hyperactivity in T1 was associated with more conduct problems in the same year. Hyperactivity in T1 had a negative influence on the slope, indicating that children with higher initial hyperactivity had a smaller reduction in their conduct problems. Gender affected the slope of conduct problems negatively, with boys having a smaller reduction in their conduct problems.

## Discussion

This study considered the association between conduct problems and hyperactivity early in life. Disruptive behaviour, as captured by the SDQ conduct problems subscale, decreased between T1 and T3 in these young children, probably as a consequence of maturation and socialisation.^33^ Hyperactivity and conduct problems are correlated. In the model where association was addressed, hyperactivity seemed to be negatively correlated with the typical decrease in conduct problems. Children with more hyperactivity at T1 had a lesser decline in conduct problems than children without hyperactivity. Hyperactivity also moderated the degree of conduct problems at the initial measuring point.

Gender was a significant predictor for the slope of conduct problems (*β* = −0.14), with boys having less reduction than girls. Adding hyperactivity to the model made the negative path even stronger (*β* = −0.25). This change in the strength of the path coefficient between gender and the slope of conduct problems when adding hyperactivity is reasonable, given that hyperactivity is more common in boys.^9^ The model still shows that both gender and hyperactivity are time-invariant predictors with roles in the development of conduct problems.

Earlier studies, using a longer time span, have indicated that hyperactivity in childhood may predict conduct problems in adolescence.^13^ Our findings corroborate this and are compatible with the assumption that hyperactivity, to a large degree, is a functional impairment, while conduct problems develop over time as a result of the interaction between predisposing characteristics such as hyperactivity and environmental influences.^15^^,^^34^ It can be argued that children with hyperactivity interact with their surroundings in a way that negatively influences the development of conduct behaviour.

Early identification of children with hyperactivity may make it possible to intervene to reduce the development of disruptive behaviour. Hyperactivity may cause problems in the child's ability to interact with both peers and adults.^35^ Not being able to take turns and wait for a response before moving to another activity causes irritation, and hyperactive children tend to be left out of peer activities and receive less attention from preschool staff (except for when they disturb group activities).^36^^,^^37^ Over time, such negative interactions may express themselves as problems in following instructions and staying in positive play interactions with peers. Children who receive more negative attention from teachers tend to have more problems with emotional regulation, concentration and disruptive behaviours.^38^ This may be one pathway for children with hyperactivity to develop more conduct problems later on.^34^ In addition, SEM analyses^35^ of the same data used in this study revealed that functional interaction with peers and preschool teachers moderated the association between hyperactivity and engagement, indicating that the negative relationship between hyperactivity and engagement can be modified by enhancing interaction with others.^39^ Engagement is known to be a strong predictor of both learning^39^ and well-being,^40^ via enhancing self-regulation.

Interventions focusing on changing negative interaction patterns between children with hyperactivity^35^ and adults and peers need to be evaluated. Interventions focusing on increasing children's engagement level will probably affect self-regulation^39^ and thus may influence hyperactivity and conduct problems exhibited by these children.

The present study has some limitations: a rather high proportion of parents did not give consent to participation, and it is possible that their children had different symptomatology compared with those included. For ethical reasons, we required informed consent from both parents, which might have contributed to the rather low participation rate. Not enough children were included longitudinally to make it possible to include other factors that might moderate and mediate outcome.

Among the children who dropped out, a higher proportion had a mother tongue other than Swedish; it is possible that these children had different symptomatology compared to those included. Another limitation may be that preschool teachers rather than parents estimated children's problem behaviour. This study was based on children in a preschool environment, and the Swedish preschool teachers providing ratings had professional knowledge (university education) of child development, and had known the specific child for at least 6 months.

Hyperactivity seems to influence the development of conduct problems, something that needs to be confirmed in future research. However, other factors such as learning and communication deficits, temperament, socioeconomic status, environmental differences and culture probably need to be added to the model to obtain a better understanding. Measures of functioning/well-being, and also interactions with parents, preschool teachers and other children, should be added in future studies. These are important factors that could moderate the link between hyperactivity and conduct behaviour problems and the subsequent development of conduct disorder.

### Conclusions

Young children's conduct problems decrease over time. Children with more initial hyperactivity have less reduction in conduct problems over time, i.e. the more hyperactivity early in life, the more conduct problems there will be later on. It is important to consider the role of hyperactivity in studies of, and intervention in, development of conduct problems.
